# Predictive Factors of Response to Selective Progesterone Receptor Modulator (Ulipristal Acetate) in the Pharmacological Treatment of Uterine Fibroids

**DOI:** 10.3390/ijerph17030798

**Published:** 2020-01-28

**Authors:** Iwona Szydłowska, Aleksandra Marciniak, Jolanta Nawrocka-Rutkowska, Aleksandra Rył, Andrzej Starczewski

**Affiliations:** 1 Department of Gynecology, Endocrinology and Gynecological Oncology, Pomeranian Medical University in Szczecin, 71-252 Szczecin, Poland; 2 Department of Medical Rehabilitation and Clinical Physiotherapy, Pomeranian Medical University in Szczecin, 71-210 Szczecin, Poland

**Keywords:** uterine myomas, ulipristal acetate, predictive factors

## Abstract

***Background***: Selective progesterone receptor modulator ulipristal acetate (UPA) is a drug used in management of symptomatic myomas. It was observed that the response to UPA treatment in uterine myomas varied amongst patients. An attempt was thus made at establishing predictive factors conducive to better reaction to treatment with UPA. The aim of this study was to assess the efficacy of UPA treatment in women with myomas, depending on pretreatment myomas’ volume, number of myomas, age of patients, estrogenic status of women, and pretreatment blood flow in uterine arteries. ***Materials and methods***: The study included patients with one to four myomas. The UPA treatment was a preparation stage for surgical treatment in all patients. The study group was divided into the subgroups according to pretreatment myomas’ volume, number of myomas, age of patients, estrogenic status of women, and pretreatment blood flow in uterine arteries. ***Results***: A better effect of reduction in size of myomas after UPA treatment was noted when pretreatment myomas’ volume was lower than 30 cm^3^. A significant reduction in fibroids’ size was observed after UPA therapy independently of the number of myomas and age of patients. A good response after the UPA therapy was observed when pretreatment estradiol concentration was below 50 pg/mL and when uterine artery resistance index (RI) was above 0.8. ***Conclusions***: Our research demonstrates that treatment with ulipristal acetate is an efficient method in preoperative preparation of patients with uterine fibroids. The most important factor of positive response to UPA therapy is myoma volume. The number of myomas and patient’s age do not interfere with effects of UPA therapy. Pretreatment estradiol concentration is significant, yet secondary for the effects of therapy. The UPA therapy has no impact on blood flow in the uterine arteries and no adverse influence on estradiol concentrations.

## 1. Introduction

Uterine leiomyomas are frequently occurring benign uterine tumors. They are typical in women at reproductive age. Symptoms associated with myomas include hypermenorrhea leading to anemia, dysmenorrhea, pelvic pain, infertility, or pregnancy complications [1,2,3].

Due to the absence of highly effective noninvasive methods of treatment, surgical techniques have remained prevalent. In recent years, more and more emphasis has been placed on conservative management of fibroids.

In 2012, selective progesterone receptor modulator (SPRM) ulipristal acetate (UPA) was registered as a new drug in management of symptomatic myomas. A mechanism of its pharmacological action is closely linked to the already well-confirmed role of progesterone in fibroid growth process. SPRMs mechanism of activity is conditioned by their binding ability with progesterone receptors. That initiates a cascade of mediators’ action and impacts on extracellular matrix (ECM), thus, finally, inhibits cell proliferation and induces apoptosis of fibroids [1,2]. The role of SPRM in reducing myomas’ size was confirmed in many cases [4,5]. SPRM affects endometrium, causing amenorrhea, and so reduces abnormal uterine bleeding associated with myomas. UPA, as other SPRMs, induces a specific progesterone receptor modulator associated with endometrial changes (PAECs), which have been described as benign and reversible [6,7]. The treatment is not associated with a decrease of estrogen concentration and side effects in the form of climacteric complaints and, subsequently, osteoporosis or cardiovascular diseases. SPRM compounds are, therefore, a very promising tool in treatment of symptomatic myoma cases.

In recent years, ulipristal acetate has been a subject of four third-phase clinical studies in the Efficacy Assessment in Reduction of Symptoms of Uterine Leiomyomata (PEARL I-IV), which proved its good bioavailability. The trials demonstrated both its efficacy in the preoperative treatment and its effectiveness and safety in a long-term treatment aiming at reduction of fibromas’ size and control of tumor-related symptoms. The trials treatment regimen consisted of daily doses of 5 and 10 mg of ulipristal acetate for 3 months or intermittent therapy of up to four courses [2,4,8,9,10].

The PEARL trials provided sufficient conclusions for the regulatory agencies of the European Union to officially approve ulipristal acetate as either a preoperative treatment of symptomatic leiomyomata or an intermittent treatment of moderate to severe symptoms of uterine fibroids in adult women at reproductive age. Esmya preparate in 5 mg tablets of ulipristal acetate was registered by the European Medicines Agency (EMA). A safe and efficient dose of one tablet a day for up to three months per one treatment course is recommended [11]. Up to this date, uliprisal acetate has not been FDA approved for treatment of symptomatic uterine leiomyomata in the United States.

Some infrequent cases of severe liver injury amongst Esmya-treated patients have been reported to medical agencies. For that reason, EMA’s Pharmacovigilance Risk Assessment Committee (PRAC), the committee responsible for evaluation of human medicines safety, considered all benefits and risks associated with Esmya usage [2,12]. PRAC concluded that the medicine must not be used in women with liver problems, however, other patients may start new treatment courses, provided they have regular liver tests.

Up to now, there is no consensus on whether the size of fibroids, their numbers, or patient’s age and estrogenic status prior to ulipristal acetate treatment affects the results of the treatment. Through observation it became apparent that the response to ulipristal acetate treatment in uterine myomas varied amongst patients. A question thus arose as to whether there were any predictive factors contributing to a possibility of a more positive reaction to treatment of fibroids with SPRM ulipristal acetate in patients.

The aim of this study was to assess the efficacy of SPRM ulipristal acetate treatment in women with myomas, depending on pretreatment volume of myomas, number of myomas, age of patients, estrogenic status of women, and pretreatment blood flow in uterine arteries.

## 2. Materials and Methods

The study was conducted at the Department of Gynecology, Gynecological Endocrinology and Oncology of the Pomeranian Medical University within a period of three years, between 2015 and 2018. A group sample consisted of thirty four patients, aged 34–55. All subjects were qualified for UPA medical treatment, as a preparation for surgical procedures.

### 2.1. Inclusion Criteria Used for the Study

- Women aged 30–55.
- Regular cycle of 25–35 days interval.
- ≥1 uterine fibroid, predominantly intramural, diagnosed by transvaginal ultrasound in diameter up to 10 cm.
- Heavy menstrual bleeding with clots.
- Participation consent.

### 2.2. Exclusion Criteria for the Study Were

- Other gynecological pathologies, except for myomas, on gynecological and ultrasound examination (e.g. uterine polyp, ovarian cyst etc.).
- Abnormal Pap smear result.
- Chronic diseases in medical history.
- Past or ongoing liver dysfunction.
- Hormonal treatment within 6 months prior to recruitment.

Some of the studied patients were undergoing a supplementation regimen with ferrum formulation, due to anemia revealed in prior laboratory tests.

All sample subjects were informed in detail about indications, benefits, and risks pertaining to the usage of ulipristal acetate in preoperative preparation.

One to four myomas ranging from 1.0 to 8.5 cm in diameter were found in all patients qualified for the study during transvaginal ultrasounds. Myoma volume was measured using an ellipsoid formula (length × width × height × 0.526). In cases with several leiomyomas, the mean volume of two to four myomas was measured. The pretreatment examinations included interviews, gynecological examinations, and ultrasounds with measurements of the amount, diameter, and volume of leiomyomas. All participating patients underwent a transvaginal color Doppler sonography with a velocimetry of the uterine arteries. The pulsatility index (PI) and resistance index (RI) were measured in left and right uterine arteries. For further analyses, both side indexes RI and PI were calculated through the mean value of both sides. Pelvic ultrasonography was performed by Voluson imaging systems with a 5 MHz probe. Scans were performed by the same ultrasonographer. Study measurements applied equal color Doppler imaging methods. Blood samples for laboratory tests were taken by vein puncture of the median cubital vein from all participating subjects in the first phase of menstrual cycle (between second and eighth day of the cycle). Estradiol concentrations were measured according to the ELISA method. Hematological examination included red cell count, hemoglobin, and hematocrit assessment.

Patients were treated with 5 mg of ulipristal acetate (Esmya; Gedeon Richter Plc.), administered orally once a day for 3 months.

A total group of thirty-four women taking part in the research was divided, according to potential predictive factors of the treatment response, into the following subgroups:

- pretreatment volume of myomas: below or above 30 cubic cm;
- number of myomas: one or more than one;
- age of patients: below or above 40
- estrogenic status of women: pretreatment estradiol concentration below or above 50 pg/mL;
- weaker or larger blood flow in uterine arteries, assessed by resistance index in the right and left uterine arteries and calculating the mean value between both sides: RI ≤ 0.8 or RI > 0.8.

Sample size calculation was strictly referring to the sample criteria, hence various size of individual sample subgroups.

The patients were monitored straight after completion of three months treatment with ulipristal acetate. The examinations included an interview, gynecological examinations, and transvaginal sonography tests with measurements of the diameter and volume of leiomyomas. A color Doppler ultrasound with a velocimetry of the uterine arteries was also performed during visits. Blood samples for laboratory tests were collected by vein puncture of the median cubital vein. As a form of a controlled post-treatment follow-up examination, estradiol concentrations and hematological parameters were measured.

Prior to the beginning of the study, the authors received the approval of the Ethics Committee of the Pomeranian Medical University in Szczecin.

### 2.3. Statistical Analysis:

Data analysis was undertaken using the Statistica for Windows v. 13,1 PL (StatSoft Inc., Tulsa, United States). The statistical analysis necessary for this study was performed through the use of the Student’s t-test. Continuous and metric variables were presented as mean ± standard deviation (SD), median (M_e),_ and minimal-maximal values. The regularity of the distribution was assessed using the Shapiro–Wilk test. Differences between groups were determined by the Mann–Whitney U-test and Student’s t-test. The statistical significance was defined as *p* < 0.05.

## 3. Results

Overall, in the entire studied group of women, a significant reduction in both volume and diameter of myomas was observed post-treatment of myomas with ulipristal acetate. In the course of three months post UPA therapy, the average fibroids volume dropped by 44.34% while the observed diameter of treated fibroids decreased by 18.32% (*p* < 0.001) (Table 1).

Due to the UPA therapy, in 7.27% of treated patients, myomas disappeared entirely.

Table 2 presents results of UPA treatment in patients with myomas’ pretreatment volume below and above 30 cubic centimeters.

A significant reduction in volume post UPA treatment was observed in all myomas, including a disappearance of myoma in one patient with myoma not larger in volume than 30 cubic centimeters after completion of three months UPA therapy. The volume of other myomas in this group was reduced significantly by 51.16% (*p* < 0.001).

In patients with myomas larger than 30 cubic centimeters, no complete disappearance of myomas was noted. The mean myoma shrinkage amounted to 19.62% (*p* < 0.001). No reduction in myomas’ volume post UPA therapy was observed in three patients (11.53%) in this group.

Table 3 presents effects of UPA treatment in patients with one or several leiomyomas.

A significant reduction in volume and diameter of myomas was also observed post-treatment with ulipristal acetate in groups of patients with one and several leiomyomas (*p* < 0.05).

Table 4 presents results of UPA treatment in women with myomas according to patients’ age, that is, below or above 40.

Volume and diameter of myomas dropped significantly, independently of patients’ age (*p* < 0.05). It has to be noted, however, that in women below 40, volume of myomas was reduced by 20%, and in those above 40, by 29.57%.

Table 5 presents effects of UPA treatment depending on pretreatment estrogenic status of women: estradiol concentration below or above 50 pg/mL.

A significant reduction in the volume and diameter of myomas was observed after the UPA therapy in the group with estradiol below 50 pg/mL (*p* < 0.05). The mean myoma shrinkage amounted to 27.45%. In patients with pretreatment estradiol above 50 pg/mL, a significant reduction in diameter was observed (*p* < 0.05), but there were no substantial volume changes in myomas (*p* > 0.05). The mean myoma shrinkage after the UPA therapy amounted to 19.7%.

Following the calculation of mean value of resistance index from the left and right uterine arteries, patients were divided according to weaker or increased blood flow in uterine arteries and the mean value of resistance index from both uterine arteries: RI ≤ 0.8 or RI > 0.8. Results of UPA therapy for both groups of patients are presented in Table 6.

UPA therapy caused not significant changes in mean PI and the RI values in the group with RI ≤ 0.8 (*p* > 0.05), and in the group with RI > 0.8, change of RI value was on the border of statistical significance.

A better response to ulipristal acetate was observed in the group with RI > 0.8, resulting in significant volume and diameter reduction of myomas post-treatment (*p* < 0.05). A group of patients with resistance index ≤ 0.8 presented with a significant reduction in diameter of post-treatment myomas (*p* < 0.05), but not noticeably significant in volume (*p* > 0.05).

UPA therapy had no impact on blood flow in uterine arteries—assessment by measurement of the PI (pulsatility index) and the RI (resistance index) (*p* > 0.05). No significant differences in the pulsatility and resistance indexes between the right and left uterine arteries before and after UPA treatment were observed (Table 7).

Treatment with ulipristal acetate had no evident influence on estradiol concentrations (*p* > 0.05) (Table 8).

The UPA therapy caused an increase in blood morphology parameters including red cell count, hemoglobin, and hematocrit. These changes occurred in all groups, divided according to the above-mentioned study parameters (*p* < 0.001) (Table 8).

No side effects, such as headaches, hot flushes, stomach aches, acne, muscle and bone pain, pelvic pain, and weight increase were observed during and post therapy with ulipristal acetate in all treated patients. Within the first two months of drug administration, 79.41% (27/34) of women within the studied group stopped menstruating.

## 4. Discussion

In recent years, treatment methods of uterine myomas have been a subject of a number of studies, and thus gave way to an ongoing professional debate. The high cost of most popular surgical treatments as well as preferences of patients have an undeniable influence on practical decisions made by physicians on a daily basis. Surgical management of fibroids, as in every case of invasive treatment, carries associated surgical risks. Additionally, some patients lean towards methods which do not involve prolonged hospitalization. They prefer methods that allow them to carry on their daily activities and work with no interruption.

Hysteroscopy is one of the presently available surgical options, and does not require prolonged hospitalization. According to the consensus statement of the Global Congress on Hysteroscopy Scientific Committee, performing one-step or multistage myomectomy in hysteroscopy is a reasonable option in case of symptomatic submucous myomas and, as such, constitutes a gold therapeutic standard. Some authors propose performing hysteroscopy even in cases of asymptomatic submucous myomas, as it creates a possibility for histopathology assessment of tissue obtained during the procedure, and the exclusion of a malignant tumor. It has to be stated that hysteroscopy is an alternative only for patients with submucous myomas. Prior to hysteroscopic procedures, medical therapy should be considered in order to reduce the size of myomas bigger than 1.5 cm, but so far the best pharmacological strategy has not been indicated [13]. 

Up to this date, a number of options have been studied regarding conservative treatment of symptomatic fibroids. Amongst those, gonadotropin-releasing hormone agonists (GnRHa) and selective progesterone receptor modulator (SPRM), in particular, have been assessed and approved for treatment of fibroids. GnRH agonists suppress estrogen release by down-regulating the hypothalamic–pituitary–gonadal axis and cause hypoestrogenemia with all its consequences and disadvantages. For that reason, the treatment was approved for a short-term preoperative use only [1,10,14].

Research concerning epigenetic alterations as a possible patomechanism of leiomyoma formation opens up a new trajectory in understanding the disease. Yang et al. paid attention to the importance of understanding abnormal epigenetic regulation in leiomyoma stem cells, hence posed a challenge to develop a safe and efficient therapeutic regimen for uterine leiomyomata [15].

More emphasis has been, however, placed on hormonal theory of myomas’ origin. As a result, selective progesterone receptor modulators were introduced in therapy. It was observed that progesterone, through a production of aberrant extracellular matrix (ECM), plays an important role in fibroids’ growth. ECM activity is conditioned by progesterone receptors A and B (PR-A, PR-B) presence in leiomyomata. In comparison with adjacent myometrium, a concentration of these receptors in myoma tissue is elevated [1]. SPRMs, binding with these receptors, cause a size reduction in fibroids by inhibiting the cell proliferation and inducing apoptosis [2]. Precise assessment of receptor concentration and proliferation indexes in myoma tissue can shed light on drug activity and performance. More advanced tissue investigation is necessary and will be a subject of further research. 

This study assessed only clinical effects of SPRM’s therapy in everyday practice, as there seem to be little literature appraising the influence of pretreatment myoma volume on effectivity of SPRM treatment. 

This study documented a significant reduction in myoma volume post UPA treatment in the entire studied group, although the effects were more noticeable when pretreatment volume was below 30 cubic centimeters.

In 2018, Yun et al. observed that an independent factor, such as number of fibroids present, can affect the fibroid volume reduction following a 3 month UPA therapy [16]. They noticed that UPA might be ineffective in cases with multiple leiomyomas. These findings were not confirmed in our study and we noted a significant reduction in fibroid volume and diameter both in patients with one and in patients with several leiomyomas.

Until now, researchers seem to have paid little attention to the age or estrogen status of patients qualified for UPA treatment. We decided to check these correlations with effectiveness of such therapy. Although fibroid volume dropped in all patients, we noted better therapeutic results when pretreatment estradiol concentrations were below 50 pg/mL and patients were above the age of 40.

Ulipristal acetate was assessed as preoperative agent for organ-sparing surgery in patients in reproductive age with fibroids, menorrhagia, and anemia in many studies. These studies confirmed that UPA usage in such cases improves blood morphology parameters and decreases total intraoperative blood loss and operative time [4,8,9,17,18]. Our study confirms a beneficial impact of short-term UPA usage on elevation of hemoglobin, hematocrit, and red cell count.

Other researchers noted that UPA not only reduces fibroids size and controls bleeding in a high percentage of women, but also significantly improves quality of patients’ lives [10,19,20]. Significant reduction in fibroid volume, fibroid-related symptoms, and quality of lives of the patients receiving both 5 mg and 10 mg of ulipristal acetate compared with placebo were previously noted [9,21,22]. The lower dose of 5 mg of the preparate was therefore officially registered as efficient by the European Medicines Agency [11]. 

UPA therapy caused no symptoms of hypoestrogenemia, and no incidences of neurovegetative adverse side effects were noted. Similar observations were made in the group sample of this study. We noted that ulipristal acetate did not decrease estradiol concentrations in the course of treatment.

UPA was registered as an effective drug in reducing bleeding and anemia, as well as fibroid volume in women who were to undergo surgery. It proved equally effective when used intermittently for longer periods of time (up to four treatment courses). In some cases, this treatment can eliminate surgical treatment entirely. 

Other conservative treatment methods used in managing myomas, such as uterine artery embolization, for instance, directly impact on blood flow in uterine arteries. The embolization causes myoma necrosis by vascular flow disorders, as well as extensive fibrotic lesions. Such methods are thus not recommended for women desiring to become pregnant in the future [23].

Although a lot of researches assessed the effects of various hormonal treatments of uterine myomas on blood flow in uterine arteries [24,25,26], there appear to be only few studies assessing the effects of SPRM influence on this blood flow [20]. A significant increase in RI value was observed in treatments with a dose of 25 mg of asoprisnil, indicating decreased uterine artery blood flow. Such adverse effects have not been observed in women treated with a dose of 10 mg of asoprisnil [20]. There was no statistically significant change in PI value pre- and post-treatment with asoprisnil, notwithstanding the dosage of SPRM. The influence of UPA usage on parameters of blood flow in uterine arteries was not researched thus far.

The above-mentioned correlations were thoroughly investigated in our study. It can be concluded that treatment of myomas with a dose of 5 mg of ulipristal acetate has no major impact on blood flow in uterine arteries. The effects of initial parameters of resistance index (RI) on response to treatment with ulipristal acetate was also examined. A better response to treatment in terms of post-treatment myomas’ size was noted, providing the preliminary RI value was >0.8. The usefulness of RI values as potential predictive factors of the treatment response might be worth re-analyzing in the context of a larger population sample. 

RI indicator can be a prognostic factor in the differentiation of malignant and benign tumors. A number of researchers observed that values of RI below 0.6 are typical for malignant neoplasms, like sarcomas and cervical cancers, but not for benign myomas [27,28,29]. In our study, the lowest RI value amounted to 0.66.

Esmya treatment is usually associated with the following side effects: amenorrhea and endometrial thickening, headaches and hot flushes, stomach aches, acne, muscle and bone pain, pelvic pain, and weight increase.

No side effects of Esmya treatment were observed in our study patients.

A relatively small study sample may pose limitations to the study findings. Additionally, due to the fact that researched mayomas were localized intramurally, leiomyomata were not assessed histopathologically, but diagnosed on the basis of ultrasonographic imagining only. 

The strength of the study lies in its straightforward applicability to the clinical context. The findings specifically point to the fact that the volume of leiomyomata can be used as a predictive factor in qualification for UPA therapy, as much better therapeutic effects are obtained in case of smaller volume tumors ≤30 cm^3^. The age of patients, pretreatment estradiol concentration, as well as parameters of pretreatment blood flow in uterine arteries seem to have secondary importance for the effects of UPA therapy.

## 5. Conclusions

1. Treatment with ulipristal acetate is an efficient method in preoperative preparation of patients with uterine myomata and beneficially affects parameters of blood morphology.
2. A better effect of UPA therapy is noted when the volume of myomas is lower than 30 cubic centimeters.
3. The number of myomas present does not interfere with overall effects of UPA therapy. The age of patients and pretreatment estradiol concentration seem to have secondary importance for the effects of UPA therapy.
4. A slightly better response to ulipristal acetate was achieved when pretreatment blood flow in uterine arteries was RI > 0.8.
5. The UPA therapy has no impact on blood flow in the uterine arteries.
6. Treatment with ulipristal acetate has no adverse influence on estradiol concentrations, causes no neurovegetative symptoms, and is well tolerated.

## Figures and Tables

**Table 1:** The diameter and volume of fibroids before and after 3 months of treatment with ulipristal acetate (*n* = 34 patients).

Fibroma’s Size	Before Treatment			After Treatment			*p*
	x ± SD	M_e_	min–max	x ± SD	M_e_	min–max	
Myoma’s volume (cubic centimeters)	54.50 ± 71.31	33.07	0.80−297.77	41.26 ± 56.58	18.70	0.00−220.50	<0.001
Myoma’s diameter (centimeters)	4.11 ± 1.84	4.04	1.15− 8.40	3.51 ± 2.05	3.30	0.00−8.40	<0.001

**Table 2:** The volume of fibroids before and after 3 months of treatment with ulipristal acetate in the group with myomas ≤30 cubic centimeters and the group with myomas >30 cubic centimeters.

Fibroma Volume	Before Treatment			After Treatment			*p*
	x ± SD	M_e_	min–max	x ± SD	M_e_	min–max	
Myoma’s volume ≤ 30 (cubic centimeters) *n* = 8	10.47 ± 8.10	8.62	0.80−25.85	6.30 ± 7.49	4.21	0.00−25.90	<0.001
Myoma’s volume > 30 (cubic centimeters) *n* = 26	93.96 ± 79.59	55.50	32.54−297.77	67.77 ± 63.14	44.61	3.77−220.50	<0.001

**Table 3:** The diameter and volume of fibroids before and after 3 months of treatment with ulipristal acetate depending on the number of myomas: one myoma or multiple myomas.

Fibroma’s Size		Before Treatment			After Treatment			*p*
		x ± SD	M_e_	min–max	x ± SD	M_e_	min–max	
Group with 1 myoma *n* = 19	Myoma’s volume (cubic centimeters)	74.02 ± 77.48	48.56	2.20−297.77	52.81 ± 50.93	37.28	0.00−191.84	0.009
	Myoma’s diameter (centimeters)	4.80 ± 1.54	4.66	1.75−8.21	4.10 ± 2.01	4.20	0.00−7.16	0.003
Group with >1 myoma *n* = 15	Myoma’s volume (cubic centimeters)	43.33 ± 66.09	18.12	0.80−256.47	33.80 ± 59.55	8.97	0.00−220.50	<0.001
	Myoma’s diameter (centimeters)	3.70 ± 1.89	3.32	1.15−8.40	3.13 ± 2.01	2.71	0.00−8.40	<0.001

**Table 4:** The diameter and volume of fibroids before and after 3 months of treatment with ulipristal acetate depending on the patients’ age. In case of multiple myomas in one patient, mean value of these 2-4 myomas was calculated.

Age		Before Treatment			After Treatment			*p*
		x ± SD	M_e_	min–max	x ± SD	M_e_	min–max	
Group ≤40 (y) *n* = 12	Myoma’s volume (cubic centimeters)	69.99 ± 80.57	52.05	2.87−297.77	45.75 ± 37.65	41.61	1.26−118.59	0.028
	Myoma’s diameter (centimeters)	4.69 ± 1.76	4.89	1.78−8.21	3.91 ± 1.70	4.27	1.29−6.90	0.003
Group >40 (y) n = 22	Myoma’s volume (cubic centimeters)	55.63 ± 61.89	33.54	3.94−220.48	45.18 ± 51.64	23.62	0.00−191.84	0.035
	Myoma’s diameter (centimeters)	4.33 ± 1.40	4.23	2.01−7.50	3.71 ± 1.76	3.49	0.00−7.16	0.005

**Table 5:** The diameter and volume of fibroids before and after 3 months of treatment with ulipristal acetate depending on the pretreatment estrogenic status of patients.

Estradiol Concentration		Before Treatment			After Treatment			*p*
		x ± SD	M_e_	min–max	x ± SD	M_e_	min–max	
Group ≤50 (pg/mL) *n* = 8	Myoma’s volume (cubic centimeters)	60.23 ± 38.68	40.55	25.85−130.56	37.39 ± 42.46	29.02	0.00−114.05	0.048
	Myoma’s diameter (centimeters)	4.53 ± 0.78	4.23	3.67−5.63	3.06 ± 1.68	3.15	0.00−4.97	0.034
Group >50 (pg/mL) *n* = 26	Myoma’s volume (cubic centimeters)	61.02 ±74.99	36.10	2.87−297.77	47.53 ± 47.97	28.99	1.26−191.84	0.124
	Myoma’s diameter (centimeters)	4.44 ± 1.68	4.46	1.78−8.21	3.98 ± 1.70	3.81	1.29−7.16	0.043

**Table 6:** The mean values of the pulsatility index (PI) and resistance index (RI) of uterine arteries before and after 3 months of treatment with ulipristal acetate depending on the initial parameters of blood flow in uterine arteries. RI and PI indexes were calculated as the mean of both sides: from left and right uterine arteries.

Parameter of Blood Flow in Uterine Arteries		Before Treatment			After Treatment			*p*
		x ± SD	M_e_	min–max	x ± SD	M_e_	min–max	
RI ≤ 0.8 *n* = 17	RI	0.73 ± 0.05	0.73	0.66−0.80	0.74 ± 0.08	0.75	0.60−0.86	0.412
	PI	1.48 ± 0.26	1.48	0.97−2.02	1.60 ± 0.34	1.59	1.08−2.08	0.635
	Myoma’s volume (cubic centimeters)	58.48 ± 71.87	38.84	4.53−297.99	41.90 ± 37.92	30.31	0.00−118.59	0.109
	Myoma’s diameter (centimeters)	4.72 ± 1.22	4.46	3.21−8.21	3.83 ± 1.65	3.93	0.00−6.90	0.003
RI > 0.8 *n* = 17	RI	0.87 ± 0.05	0.87	0.81−1.00	0.84 ± 0.06	0.84	0.67−0.91	0.049
	PI	2.16 ± 0.32	2.10	1.73−2.84	2.10 ± 0.44	2.02	1.26−2.84	0.778
	Myoma’s volume (cubic centimeters)	63.08 ± 67.06	38.11	2.87−220.48	48.67 ± 54.18	28.62	1.26−191.84	0.006
	Myoma’s diameter (centimeters)	4.22 ± 1.77	4.06	1.78−7.50	3.75 ± 1.82	3.21	1.29−7.16	0.005

**Table 7:** The mean values of the PI and RI of the right and left uterine arteries before and after 3 months of treatment with ulipristal acetate (*n* = 34 patients).

Parameter of Blood Flow in Uterine Arteries		Before Treatment			After Treatment			*p*
		x ± SD	M_e_	min–max	x ± SD	M_e_	min–max	
PI	Right uterine artery	1.83 ± 0.49	1.82	0.82−2.78	1.87 ± 0.62	1.71	0.78−3.79	0.546
	Left uterine artery	1.81 ± 0.57	1.70	0.83−3.29	1.84 ± 0.55	1.77	0.94−3.33	0.692
RI	Right uterine artery	0.80 ± 0.09	0.81	0.60−1.00	0.79 ± 0.10	0.80	0.52−0.95	0.788
	Left uterine artery	0.80 ± 0.10	0.79	0.59−1.00	0.80 ± 0.09	0.80	0.64−0.96	0.553

**Table 8:** Hormonal and hematological parameters of blood serum before and after 3 months of fibroids treatment with ulipristal acetate (*n* = 34 patients).

Hormonal and Hematological Parameters	Before Treatment			After Treatment			*p*
	x ± SD	M_e_	min–max	x ± SD	M_e_	min–max	
RBC (mln/uL)	4.39 ± 0.43	4.31	3.21−5.38	4.69 ± 0.35	4.72	3.93−5.48	<0.001
Hb (g/dL)	12.01 ± 1.80	12.25	8.10−15.40	13.13 ± 1.13	13.50	10.30−15.30	<0.001
Ht (%)	36.04 ± 4.66	36.65	25.40−43.50	38.82 ± 3.19	38.85	31.30−44.90	<0.001
E2 (pg/mL)	16.50 ± 83.55	94.86	6.95−387.60	110.78 ± 108.83	65.07	7.70−470.60	0.846

RBC—red cell count, Hb—hemoglobin, Ht—hematocrit, E2—estradiol.

## Abbreviations

UPA	Ulipristal acetate
SPRM	Selective progesterone receptor modulator
PAECs	Progesterone receptor modulator-associated endometrial changes
PI	Pulsatility index
RI	Resistance index
ECM	Extracellular matrix
GnRHa	Gonadotropin-releasing hormone agonists
PRAC	Pharmacovigilance Risk Assessment Committee
